# Analysis of the lung microbiota in dogs with *Bordetella bronchiseptica* infection and correlation with culture and quantitative polymerase chain reaction

**DOI:** 10.1186/s13567-020-00769-x

**Published:** 2020-03-24

**Authors:** Aline Fastrès, Morgane A. Canonne, Bernard Taminiau, Frederic Billen, Mutien-Marie Garigliany, Georges Daube, Cécile Clercx

**Affiliations:** 1 Department of Clinical Sciences, FARAH, Faculty of Veterinary Medicine, University of Liège, 4000 Liège, Belgium; 2 Department of Food Sciences-Microbiology, FARAH, Faculty of Veterinary Medicine, University of Liège, 4000 Liège, Belgium; 3 Department of Veterinary Pathology, FARAH, Faculty of Veterinary Medicine, University of Liège, 4000 Liège, Belgium

## Abstract

Infection with *Bordetella bronchiseptica* (*Bb*), a pathogen involved in canine infectious respiratory disease complex, can be confirmed using culture or qPCR. Studies about the canine lung microbiota (LM) are recent, sparse, and only one paper has been published in canine lung infection. In this study, we aimed to compare the LM between *Bb* infected and healthy dogs, and to correlate sequencing with culture and qPCR results. Twenty *Bb* infected dogs diagnosed either by qPCR and/or culture and 4 healthy dogs were included. qPCR for *Mycoplasma cynos* (*Mc*) were also available in 18 diseased and all healthy dogs. Sequencing results, obtained from bronchoalveolar lavage fluid after DNA extraction, PCR targeting the V1–V3 region of the 16S rDNA and sequencing, showed the presence of *Bb* in all diseased dogs, about half being co-infected with *Mc*. In diseased compared with healthy dogs, the β-diversity changed (*P* = 0.0024); bacterial richness and α-diversity were lower (*P* = 0.012 and 0.0061), and bacterial load higher (*P* = 0.004). *Bb* qPCR classes and culture results correlated with the abundance of *Bb* (r = 0.71, *P* < 0.001 and r = 0.70, *P* = 0.0022). *Mc* qPCR classes also correlated with the abundance of *Mc* (r = 0.73, *P* < 0.001). *Bb* infection induced lung dysbiosis, characterized by high bacterial load, low richness and diversity and increased abundance of *Bb*, compared with healthy dogs. Sequencing results highly correlate with qPCR and culture results showing that sequencing can be reliable to identify microorganisms involved in lung infectious diseases.

## Introduction

*Bordetella bronchiseptica*, a Gram-negative, aerobic, coccobacillus, is regarded as one of the principal pathogens involved in canine infectious respiratory disease complex (CIRD-C) [1–4]. Its prevalence in dogs with infectious respiratory diseases ranges from 5.2 to 78.7% [2, 4–6]. According to the taxonomical classification, the bacterium *B. bronchiseptica* belongs to the Proteobacteria phylum, the Alcaligenaceae family and the *Bordetella* genus [7]. CIRD-C or formerly “kennel cough” is considered as one of the most common infectious diseases in dogs worldwide despite vaccination, and affects mostly young and kennel dogs [8]. Viruses such as canine adenovirus, canine distemper virus, canine parainfluenza virus, canine respiratory coronavirus, pneumovirus and influenza A virus and bacteria other than *B. bronchiseptica* such as *Mycoplasma cynos* and *Streptococcus equi* subsp. *zooepidermicus* are primary infectious agents involved in the complex [4, 8]. Because of the numerous infectious etiologies as well as possible co-infections, clinical signs of CIRD-C are highly variable and difficult to predict ranging from mild illness to severe pneumonia or death [8]. Among the bacteria, *Mycoplasma cynos*, a Gram-negative organism is considered as an emerging bacterium in CIRD-C [4, 9]. This bacterium belongs to the Tenericutes phylum, the Mycoplasmataceae family and the *Mycoplasma* genus [10]. The diagnosis of *B. bronchiseptica* infection can be confirmed either by culture or by specific quantitative polymerase chain reaction (qPCR) on various samples including bronchoalveolar lavage fluid (BALF). The bacteria can also be observed on cytological preparations, adhering to the top of the cilia of respiratory epithelial cells [1]. The treatment against *B. bronchiseptica* can be challenging as the bacterium is localized at the top of the cilia, can adopt a biofilm lifestyle and may drive an immunosuppressive response [11–15]. In such cases, classical oral or parenteral antimicrobial drug may not be sufficient even if in vitro susceptibility is shown [16]. Recently, it has been shown that gentamycin nebulization was helpful to achieve therapeutic concentration on the apical surface of bronchial epithelium, mostly when classical antimicrobial drugs failed to be curative [17–19].

The 16S rDNA amplicon sequencing is a technique less sensitive than a qPCR but which allows rapid and accurate identification of all the bacteria composing the microbiota, which refers to the global microbial population of an area, including rare, unknown, slow-growing and unculturable bacteria [20–23]. Moreover, this technique allows to highlight the complexity of the microbial populations and their alterations in disease processes [20, 23]. In man, the 16S rDNA amplicon sequencing is increasingly being used in clinical contexts such as in acute pneumonia. Acute pneumonia is considered as an abrupt, emergent phenomenon with the predominance of specific taxonomic groups, low microbial diversity and high bacterial load [24–26]. Studies in acute pneumonia indicate that the 16S rDNA sequencing improves the microbiological yield and could help to guide antimicrobial therapy [20, 27]. In dogs, the lung microbiota (LM) has only been studied in bacterial secondary or community-acquired pneumonia (CAP) and only few data are available in experimental healthy beagles [28–30] and healthy dogs from other breeds [31]. In dogs with pneumonia, a dysbiosis of the LM was observed with the loss of bacteria found in health and the domination, mostly in CAP, of one or two bacteria [30]. Moreover a good agreement was found between the results of 16S rDNA amplicon sequencing and culture, although some discrepancies concerning the number of unique taxa identified and presence or absence of predominating taxa were noticed [30]. Results suggest that the 16S rDNA amplicon sequencing could be useful for causal bacteria detection in parallel with culture, mostly if culture is negative [30].

The aims of this study were to analyze the LM in a series of cases with *B. bronchiseptica* infection in comparison with healthy dogs and to correlate results of the 16S rDNA amplicon sequencing with qPCR and culture results.

## Materials and methods

### Case selection criteria

Client-owned dogs referred to the veterinary hospital of the University of Liège, between January 2014 and December 2018, with a diagnosis of *B. bronchiseptica* infection, were recruited. Infection with *B. bronchiseptica* was confirmed by either positive culture (> 10^4^ colony forming unit/mL), or positive qPCR, or both, on BALF samples and by the resolution of the clinical signs after adapted antimicrobial drug administration. Another inclusion criterion concerned the availability of BALF banked at −80 °C, for LM analysis. Data were collected from the medical records and included signalment, history, clinical signs, thoracic radiography, bronchoscopy findings and BALF analysis results, as well as culture and qPCR results.

BALF samples from healthy dogs involved in an independent study analyzing the effect of the type of breed on the LM composition were also used. Those samples were obtained according to a protocol approved by the Ethical Committee of the University of Liège (protocol #1435) and after the owner consent. Healthy status was confirmed based on a complete history without abnormalities, normal physical examination, blood work (hematology and biochemistry), bronchoscopy and BALF analysis (gross appearance and cell counts). Healthy dogs did not receive any kind of antimicrobial drugs or probiotics for the year preceding the study.

### BALF collection and processing

Bronchoscopy, bronchoalveolar lavage (BAL) procedure, and BALF processing and analysis were performed as already described [1, 29]. Briefly, dogs were anesthetized using various protocols at the discretion of a board-certified anesthesiologist. A flexible pediatric endoscope (FUJINON© Paediatric Video-Bronchoscope EB-530S) cleaned and disinfected before each use was inserted into the trachea until the extremity was wedged into the bronchi. Three to four mL/kg of sterile saline solution (NaCl 0.9%) divided into three aliquots were instilled into at least two different lung lobes, followed by aspiration by gentle suction. The recovered BALF was pooled. Before each BAL in dogs, a procedural control specimen (PCS) was obtained by injection and aspiration of 10 mL of sterile saline solution (NaCl 0.9%) through the bronchoscope.

Just after BALF collection, total (TCC) and differential cells counts (DCC) were determined using respectively a hemocytometer and a cytospin preparation (centrifugation at 221 *g*, for 4 min at 20 °C, Thermo Shandon Cytospin©4), by counting a total of 200 cells at high power field. Part of the crude BALF was promptly stored in cryotubes at −80 °C for the microbiota analysis and the remaining BALF was centrifuged at 3500 × *g* 15 min at 4 °C and divided into pellets and supernatant also stored separately at −80 °C. The PCSs were stored in cryotubes at −80 °C without processing.

### Culture

Culture from crude fresh BALF samples were performed for aerobic bacteria detection. Cultures were conducted at 35 °C on several agar plates (Chapman’s, Mac Conkey’s, CAN and TSS agar). Standard biochemical methods were used to identified the bacteria (Synlab Laboratories, Liège, Belgium). Due to challenging growth requirements and as it is not classically performed in clinic, *Mycoplasma* sp. was not cultured. BALF samples from healthy dogs were not submitted to conventional bacterial culture.

### *B. bronchiseptica* and *M. cynos* qPCR

In diseased dogs, qPCR targeting *B. bronchiseptica* and *M. cynos* were performed either on crude fresh BALF when performed immediately after the BAL procedure or on pellet and crude frozen BALF when performed later. In healthy dogs, qPCRs were performed on frozen pellet BALF (Department of Veterinary Pathology, Liège, Belgium).

DNA was extracted from samples using the NucleoMag Vet kit (Macherey-Nägel GmbH & Co. KG, Düren, Germany) according to the protocol provided by the manufacturer. Total DNA quantity and purity were measured after extraction using the ND-1000 spectrophotometer (NanoDrop ND-1000, Isogen, De Meern, The Netherlands).

For *B. bronchiseptica* and *M. cynos* detection, duplicate qPCR reactions (20 µL) included 2 µL of DNA template, 10 µL Luna Universal Probe qPCR Master Mix (Bioké, The Netherlands), 6 µL of water and 2 µL of the primers mix. For *B. bronchiseptica*, the primers mix contained 20 µL of the forward primer (5′-ACTATACGTCGGGAAATCTGTTTG -3′) and the reverse primer (5′-CGTTGTCGGCTTTCGTCTG -3′) at 10 µM and 10 µL of the probe (5′-FAM-CGGGCCGATAGTCAGGGCGTAG-BHQ1-3′) at 10 µM [32]. The cycling conditions started with an initial denaturation step at 95 °C for 10 min, followed by 45 cycles of denaturation at 95 °C for 30 s, primer annealing at 55 °C for 20 s and elongation at 72 °C for 1 min. For *M. cynos,* the primers mix contained 20 µL of the forward primer (5′-GTGGGGATGGATTACCTCCT-3′) and the reverse primer (5′-GATACATAAACACAACATTATAATATTG-3′) at 10 µM and 10 µL of the probe (5′-TCTACGGAGTACAAGTTACAATTCATTTTAGT-3′) at 10 µM [33]. The cycling conditions were as follows: an initial denaturation step at 95 °C for 10 min, followed by 45 cycles of denaturation at 95 °C for 30 s, primer annealing at 50 °C for 20 s and elongation at 72 °C for 1 min.

Results obtained were further categorized into 6 classes for the correlation with the LM calculation according to a previously published study [1]. Briefly, classes were defined based on the cycle threshold (Ct) values: very high load (Ct < 20), high load (20.1–24), moderate load (24.1–28), low load (28.1–32), very low load (> 32.1), and negative results.

### 16S rDNA amplicon sequencing

Analysis of the LM in all samples was performed for each step (DNA extraction, polymerase chain reactions (PCRs), sequencing and post sequencing analysis) on a single occasion for all samples. As required, strict laboratory controls were done to avoid contaminations from PCR reagents and laboratory materials.

DNA was extracted from crude BALFs and PCSs previously banked at −80 °C, following the protocol provided with the DNeasy Blood and Tissue kit (QIAGEN Benelux BV; Antwerp, Belgium) as already described [29, 34]. Total DNA quantity and purity were measured after extraction using the ND-1000 spectrophotometer (NanoDrop ND-1000, Isogen, De Meern, The Netherlands).

Duplicate qPCRs targeting the V2-V3 region of the 16S rDNA were performed to evaluate the bacterial load in the lung as already described [29, 35]. qPCRs were conducted in a final volume of 20 µL containing 2.5 μL of template DNA, 0.5 μL of forward primer (5′-ACTCCTACGGGAGGCAGCAG-3′; 0.5 μM), 0.5 μL of reverse primer (5′-ATTACCGCGGCTGCTGG-3′; 0.5 μM), 10 μL of No Rox SYBR 2 × MasterMix (Eurogentec, Seraing, Belgium), and 6.5 μL of water. Data were recorded using an ABI 7300 real-time PCR system, with the following cycling sequence: 1 cycle of 50 °C for 2 min; 1 cycle of 95 °C for 10 min; 40 cycles of 94 °C for 15 s; and 1 cycle of 60 °C for 1 min. A melting curve was constructed in the range of 64–99 °C and the end of the cycle. The run contained also non-template controls and a tenfold dilution series of a V2–V3 PCR product purified (Wizard^®^ SV Gel and PCR Clean-Up System, Promega, Leiden, The Netherlands), quantified by PicoGreen targeting double-stranded DNA (Promega) and used to build the standard curved. The results reflecting the bacterial load were expressed in logarithm with base 10 of the copy number per milliliter.

To characterize the bacterial populations in samples, the V1–V3 region of the bacterial 16S rDNA gene was amplified using the forward primer (5′-GAGAGTTTGATYMTGGCTCAG-3′) and the reverse primer (5′-ACCGCGGCTGCTGGCAC-3′) with Illumina overhand adapters as previously described [29, 34]. PCRs were conducted and amplicons obtained purified with the Agencourt AMPure XP beads kit (Beckman Coulter, Villepinte, France), indexed using the Nextera XT index primers 1 and 2 and quantified by PicoGreen (ThermoFisher Scientific, Waltham, MA, USA) before normalization and pooling to form libraries. The amplification products < 1 ng/µL were not sequenced.

Sequencing were performed on a Miseq Illumina sequencer using V3 reagents with positive controls and negative controls from the PCR step.

A total of 3 254 346 reads were obtained after sequencing with a median length of 510 nucleotides. After a first cleaning step, 3 116 730 reads were screened for chimera using Vsearch [36]. 3 040 049 reads were retained for alignment and clustering using MOTHUR v1.40 [37]. The taxonomical assignation with an operational taxonomic unit (OTU) clustering distance of 0.03 were based on the SILVA database v1.32. A final subsampling was performed with a median reads per samples of 10 000 reads.

### Statistical analyses

To compare diseased and healthy dogs, a subpopulation of dogs with *B. bronchiseptica* infection was selected to be age-matched with the population of healthy dogs (Mann–Whitney tests using XLStat software).

Normality was checked with Shapiro–Wilk tests before each comparison between healthy and diseased dogs. Mann–Whitney tests were used to compared TCC and DCC between diseased and healthy dogs using XLStat software. Differences in relative abundances between groups at all the taxonomic levels were assessed by Welch’s t-tests and Benjamini–Hochberg–false discovery rate of 10% correction [38], with STAMP software. The β-diversity was evaluated by a permutational analysis of the variance (PERMANOVA) and visualized with a principal component analysis (PCA) using R (R vegan package). Other ecological parameters of the LM were calculated using MOTHUR v1.40 and compared between healthy and diseased dogs with Mann–Whitney tests using XLStat software. The α-diversity was based on the inverse Simpson index, the richness on the chao index and the evenness was derived from the Simpson index. The bacterial load was compared between groups with Mann–Whitney tests using XLStat software. The bacterial load in PCSs were compared with the corresponding bacterial load in BALF samples with a Wilcoxon signed-rank test using XLStat software.

Correlations between the lung bacterial communities at each taxonomic level and the Ct classes for either *B. bronchiseptica* or *M. cynos*, and the culture results, were measured with Spearman tests using XLStat software.

Data were expressed as median and interquartile range. A *P* value < 0.05 was considered as statistically significant.

## Results

### Animals

Twenty dogs with a diagnosis of *B. bronchiseptica* infection and 4 healthy dogs were included in the study (Table 1). In all dogs, median age was 9 months (range 3-18) and medium weight was 11.5 kg (1.3–41.0). From the 20 diseased dogs, seven (dogs no. 3, 9, 14, 15, 18, 19 and 20) were selected and compared with the 4 healthy dogs. No significant difference in the age was found between the subpopulation of diseased dogs and the healthy dogs (*P* = 0.073). For the TCC, DCC and all LM parameters (including relative abundances at all taxonomic levels, the bacterial load and the ecological parameters including the β and α-diversity, the richness and the evenness), differences between the subpopulations of diseased dogs selected or not for the comparison with healthy dogs were not significant indicating that the subsampling is representative of all the diseased group (see Additional file 1).

**Table 1:** **Characteristic of the dogs included in the study**

Dogs	Status	Age at sampling (years)	Sex	Breed	Antibiotic treatment at sampling	Ct *B. bronchiseptica*	Ct *M. cynos*	Culture
1	Diseased	0.60	M	French bulldog	−	28.4	22.9	/
2	Diseased	0.40	M	Malamute	+ (amoxicillin/clavulanic acid 12.5 mg/kg BID and enrofloxacin 5 mg/kg SID, PO, for 1 day)	22.3	18.5	−
3	Diseased	1.05	F	French bulldog	−	25.3	−	+ (*B. bronchiseptica*)
4	Diseased	0.43	F	Boxer	−	21.9	−	+ (*B. bronchiseptica*)
5	Diseased	0.65	F	French bulldog	+ (doxycycline 5 mg/kg BID, PO, for 10 days)	24.2	LOD	+ (*B. bronchiseptica*)
6	Diseased	0.32	F	English bulldog	+ (marbofloxacin 3 mg/kg SID, PO, for 7 days)	?	?	−
7	Diseased	0.35	F	Jack Russel terrier	−	/	/	+ (*B. bronchiseptica*)
8	Diseased	0.54	M	Boxer	+ (amoxicillin/clavulanic acid 12.5 mg/kg BID, PO for 1 day)	26.5	24.0	–
9	Diseased	0.99	F	Munster lander	−	23.6	−	+ (*B. bronchiseptica*)
10	Diseased	0.38	F	French bulldog	−	?	/	/
11	Diseased	0.56	F	Chihuahua	−	24.0	?	−
12	Diseased	0.51	F	Cavalier king Charles spaniel	−	24.0	−	+ (*B. bronchiseptica*)
13	Diseased	0.57	F	German shepherd	−	21.5	LOD	+ (*B. bronchiseptica*)
14	Diseased	0.68	F	Cavalier king Charles spaniel	−	25.6	23.7	+ (*B. bronchiseptica*, *Acinetobacter baumanii*)
15	Diseased	0.99	M	Spitz	−	17.6	−	+ (*B. bronchiseptica*)
16	Diseased	0.53	M	Boxer	−	25.3	32.8	/
17	Diseased	0.27	M	Yorkshire terrier	−	21.8	−	+ (*B. bronchiseptica*)
18	Diseased	0.90	F	Spitz	−	23.5	−	+ (*Pantoea agglomerans*, *Serratia marcescens*)
19	Diseased	1.51	M	Chinese crested	+ (doxycycline, one injection, dose unknown)	32.0	−	−
20	Diseased	0.72	M	Cavalier king Charles spaniel	−	21.5	−	+ (*B. bronchiseptica*)
21	Healthy	1.26	M	Beauceron	−	−	−	/
22	Healthy	1.19	M	French bulldog	−	−	−	/
23	Healthy	1.40	M	French bulldog	−	38.0	−	/
24	Healthy	1.43	M	Pug	−	−	−	/

qPCR, quantitative polymerase chain reaction; Ct, cycle threshold value; +, positive result; −, negative result; ?, positive qPCR result but Ct value not known;/, test not performed; LOD, only one replicate was above the detection’s limit; SID, once a day; BID, twice a day; PO, oral administration.

French bulldogs, boxers and Cavalier King Charles spaniels were among the most represented breeds and counted for 50% of the recruited dogs affected with *B. bronchiseptica*. Chronic productive daily cough of at least 1 week to 4 month’s duration (median of 1 month) was reported in all diseased cases. At presentation, 5 dogs were receiving oral antimicrobial agents (Table 1) without improvement including amoxicillin/clavulanic acid (*n* = 1), amoxicillin/clavulanic acid with enrofloxacin (*n* = 1), doxycycline (*n* = 2) and marbofloxacin (*n* = 1). Vaccinal status was recorded for 15 dogs, 6 dogs were not vaccinated against *B. bronchiseptica* and 9 received only one subcutaneous vaccinal injection (Pneumodog©, Merial, Lyon, France) between one and 12 months (median 2 months) before the development of symptoms. Physical examination was normal in 5 dogs, positive laryngo-tracheal reflex was noted in 10 dogs, 5 dogs had bilateral nasal discharge, 2 had dyspnea and 1 had mild hyperthermia (39.1 °C). Thoracic radiography revealed the presence of a ventral alveolar pattern in 9 dogs, a broncho-interstitial pattern in 8 dogs and no abnormalities in 3 dogs. The diagnosis of *B. bronchiseptica* infection was confirmed by a positive qPCR (*n* = 9), a positive culture (*n* = 1) or both (*n* = 10).

### Bronchoscopy and BALF analysis

During the bronchoscopy procedure, in diseased dogs, mucopurulent material was seen in the trachea and bronchi in 14 dogs, edema and/or erythema and/or thickening of the bronchial wall was noted in 10 dogs, bronchomalacia was reported in 4 dogs. TCC and DCC were available in the BALF of 18 and 17 diseased dogs, respectively. In all the diseased dogs, median TTC was 1740 cells/µL (1080–3515) and the median differential cell count included 39% (12–63) of macrophages, 41% (24–77) of neutrophils, 7% (4–12) of lymphocytes and 1% (0–5) of eosinophils.

Compared with healthy dogs, the TTC in the subpopulation of dogs affected with *B. bronchiseptica* was significantly higher with more neutrophils and less macrophages (Table 2).

**Table 2:** **Total and differential cell counts between the subpopulation of diseased dogs and healthy dogs**

	Total cell count (cells/µL)	Macrophages (%)	Neutrophils (%)	Lymphocytes (%)	Eosinophils (%)
Subpopulation of diseased dogs (*n* = 7)	1300 (1040–3622)	33 (15.8–47.2)	48 (35.8–68.5)	9.5 (6.2–15.8)	2 (1–6.8)
Healthy dogs (*n* = 4)	270 (243.8–380)	91.5 (85.5–96.2)	2.5 (2–3.2)	6 (0.8–11.2)	0.5 (0–1.5)
*P*-value	*0.0061*	*<0.001*	*0.014*	0.29	0.32

Results are expressed as median (range). Significant *P*-value are in italic. The subpopulation of diseased dogs corresponds to the dogs no. 3, 9, 14, 15, 18, 19 and 20 in the Table 1.

### Culture results

In the diseased dogs, the result of the culture was positive for *B. bronchiseptica* in 6/11 dogs (54.5%) and negative in 5 dogs from which 4 were under antimicrobial treatment.

### *B. bronchiseptica* and *M. cynos* quantitative PCR

All qPCR results were positive for *B. bronchiseptica* in the diseased dogs (19/19) and included 1 very high load result, 9 high load results, 5 moderate load results and 2 low load results, one of them corresponding to a dog receiving doxycycline. Two qPCRs were positive without information available on the Ct level. qPCR results for *M. cynos* were positive in 7/18 (38.9%) and included 1 very high load result, 3 high load results and 1 very low load result. Two qPCR results were positive but Ct values were unknown.

In the healthy group, one dog had a positive qPCR result for *B. bronchiseptica* at a very low load, while the results were negative in the 3 other dogs. qPCRs for *M. cynos* were all negative in the healthy group.

### Microbiota analysis

The PCSs were not sequenced as their amplification products after purification were < 1 ng/µL. An internal study performed in our laboratory (Taminiau B and Daube G, unpublished observations) showed that under this value, the sequencing is not reliable. Moreover, the bacterial load was about 100 times lower in the PCSs compared with the samples (*P* = 0.016).

**Figure 1:**
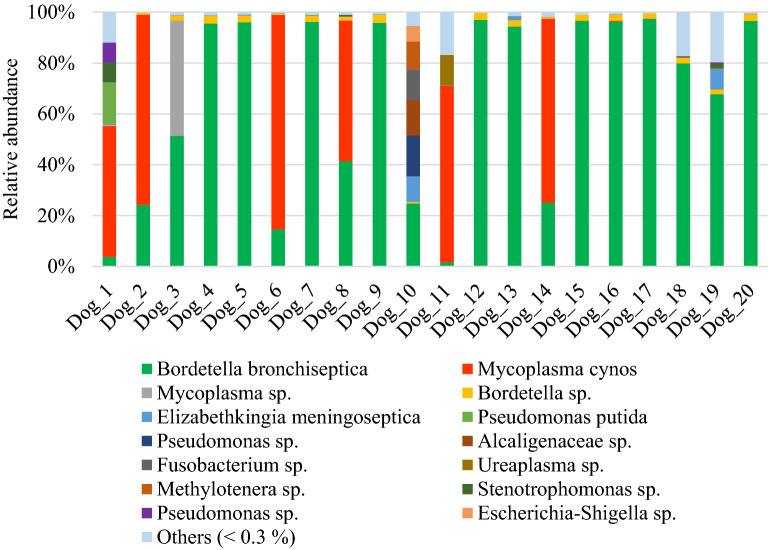
**Species-level composition of the lung microbiota in dogs affected with*B. bronchiseptica***. Bar charts showing relative abundance annotated to the taxonomic level of species of all taxa detected in the bronchoalveolar lavage fluid of 20 dogs affected with *B. bronchiseptica.*

*B. bronchiseptica* was found in each of the 20 diseased dogs with a relative abundance of more than 50% in 13 of them. Only 2 dogs (dogs n°1 and 11) had a relative abundance of *B. bronchiseptica* of less than 5% (Figure 1). Among the diseased dogs, 40% (8/20) were co-infected with *M. cynos* and/or *Pseudomonas* sp. and other strain of *Mycoplasma* than *M. cynos.* Other bacteria were also found in high relative abundance (> 5%) including, *Elizabethkingia meningoseptica*, *Stenotrophomonas* sp., *Ureaplasma* sp., Alcaligenaceae_genus sp., *Elizabethkingia meningoseptica*, *Fusobacterium* sp., *Methylotenera* sp. and *Escherichia*-*Shigella* sp. (Figure 1).

In healthy dogs, *B. bronchiseptica* was found by amplicon sequencing in one dog and *M. cynos* in 3 dogs in a very low relative abundance (0.43 and 0.55, 0.52 and 0.61% respectively).

**Figure 2:**
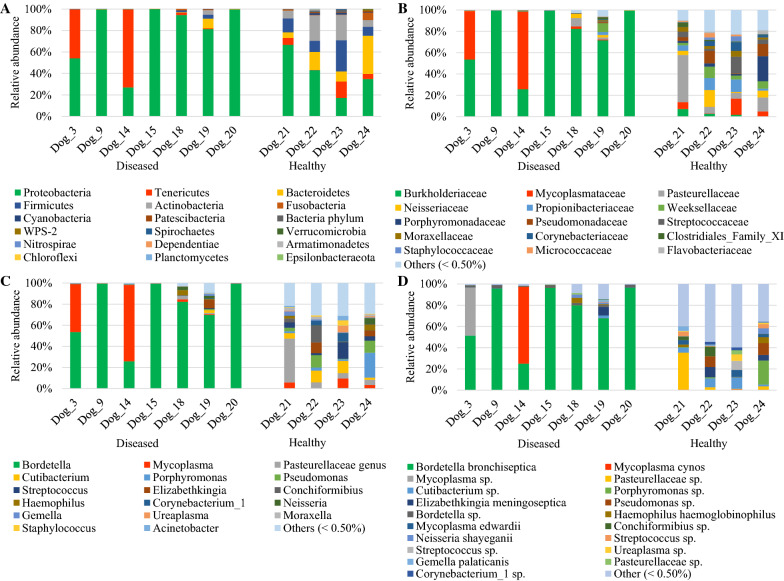
**Taxa detected in healthy dogs and dogs affected with*B. bronchiseptica***. Bar charts showing the relative abundance of all taxa detected in the bronchoalveolar lavage fluid of 4 healthy dogs and 7 dogs affected with *B. bronchiseptica*, annotated to the taxonomic level of phylum (**A**), family (**B**), genus (**C**) and species (**D**).

**Figure 3:**
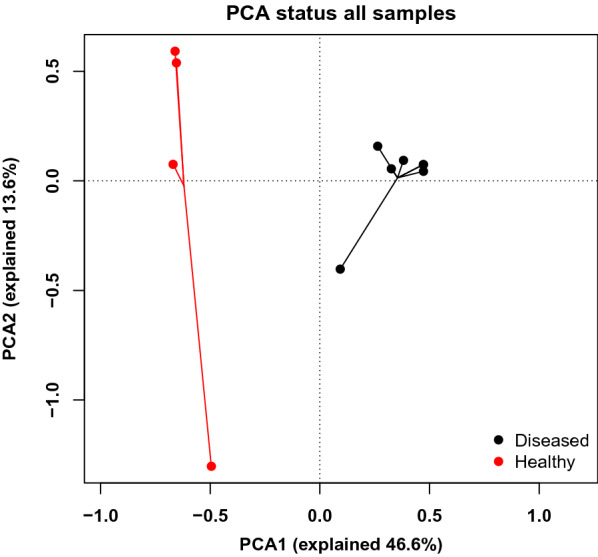
**Principal component analysis representing the β-diversity between healthy dogs and dogs affected with*B. bronchiseptica***. Lung communities are clustered by groups (diseased (*n* = 7) and healthy (*n* = 4) dogs).

**Figure 4:**
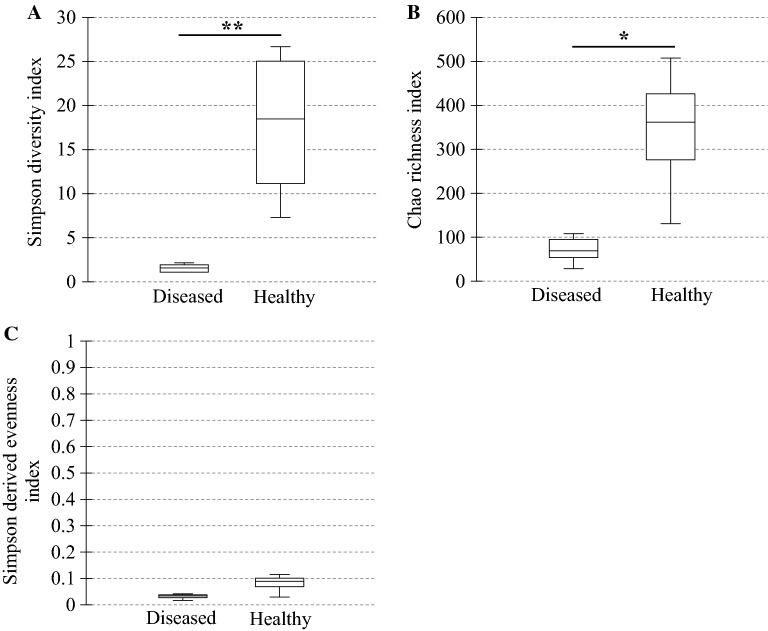
**Ecological parameters comparison between healthy dogs and dogs affected with*B. bronchiseptica***. Box plot graphs representing the bacterial alpha diversity (**A**), richness (**B**) and evenness (**C**) in healthy (*n* = 4) compared with diseased dogs (*n* = 7). The medians are represented by the central horizontal bars. The lower and upper limits of the box are the first and third quartiles, respectively. *P = 0.012; **P = 0.006.

**Figure 5:**
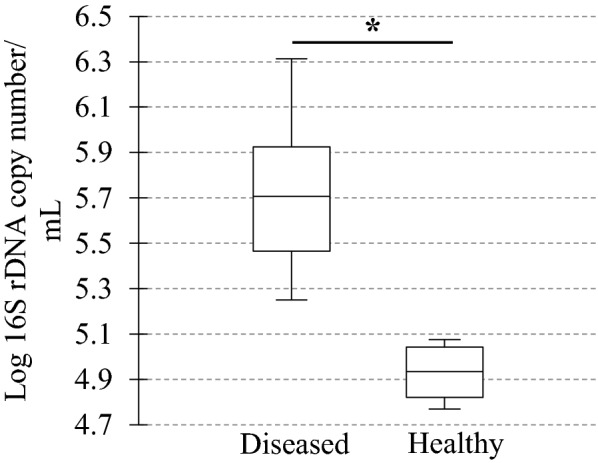
**Bacterial load in healthy dogs and dogs affected with*B. bronchiseptica***. Box plot representing the logarithm of the number of 16S rDNA copies per microliter (bacterial load) between healthy (*n* = 4) and diseased dogs (*n* = 7). The medians are represented by the central horizontal bars. The lower and upper limits of the box are the first and third quartiles, respectively. *P = 0.004.

In diseased compared with healthy dogs, a shift was observed in the bacterial populations with more Alcaligenaceae in diseased compared with healthy dogs (82.3% (62.6-99.4) versus 2.2% (1.3–3.8); *P*-value corrected = 0.058) at the family level (Figure 2B). At the genus level (Figure 2C), there were more *Bordetella* in diseased compared with healthy dogs (82.3% (61.7-99.4) versus 0% (0–0.1); *P*-value corrected = 0.11). There was no significant difference at the phylum (Figure 2A) and at the species levels (Figure 2D), although a marked increase in Proteobacteria (94.3% (67.6-99.6) versus 38.9% (30.4–49.0); *P*-value corrected = 0.30) phylum reflecting the increase in *B. bronchiseptica* (79.8% (59.5–96.2) versus 0% (0–0.1); *P*-value corrected = 0.40) species was noted in diseased compared with healthy dogs. The β-diversity (Figure 3) assessed by the PERMANOVA was significantly different between healthy and diseased dogs (*P* = 0.0024). The α-diversity (Figure 4A) as well as the richness (Figure 4B) were significantly lower in diseased compared with healthy dogs. There was no difference between healthy and diseased dogs for the evenness (*P* = 0.10) ((Figure 4C). Finally, the bacterial load was significantly higher in dogs with *B. bronchiseptica* infection compared with healthy dogs (Figure 5).

A significant positive correlation was found between the bacterial composition in *B. bronchiseptica* and *M. cynos* at each taxonomic level obtained by the 16S rDNA amplicon sequencing and the Ct classes for *B. bronchiseptica* and *M. cynos*, and the culture results as shown in Table 3. In all cases where a positive culture was found for *B. bronchiseptica*, the relative abundance for *B. bronchiseptica* was highly elevated (96.01% (94.87–96.56)). In 2 dogs, other bacteria were identified by culture including *Acinetobacter baumanii*, *Pantoea agglomerans* and *Serratia marcescens* (Table 1) but were not identified by sequencing.

**Table 3:** **Correlation between the 16S rDNA amplicon sequencing and either Ct classes or culture**

16S rDNA amplicon sequencing results	*B. bronchiseptica*				*M. cynos*	
	qPCR results		Culture results		qPCR results	
	r	*P*-value	r	*P*-value	r	*P*-value
Phyla						
Proteobacteria	0.54	0.012	0.70	0.0022		
Tenericutes					0.66	0.0018
Families						
Alcaligenaceae	0.66	0.0015	0.70	0.0021		
Mycoplasmataceae					0.66	0.0018
Genera						
Bordetella	0.73	< 0.001	0.70	0.0022		
Mycoplasma					0.66	0.0018
Species						
Bordetella_bronchiseptica	0.71	< 0.001	0.70	0.0022		
Mycoplasma_cynos					0.73	< 0.001
Bordetella_Otu00473	0.70	< 0.001	0.68	0.0017		

Significant positive correlation results between the bacterial composition of the LM in all dogs (*n* = 24) for *B. bronchiseptica* and *M. cynos* at each taxonomic level and either *B. bronchiseptica* and *M. cynos* Ct classes or the culture. qPCR, quantitative polymerase chain reaction; r, Spearman correlation coefficient

## Discussion

In the present study, we described the LM in dogs with CIRD-C and *B. bronchiseptica* infection. We showed a clear dysbiosis of the LM with a significant decrease in α-diversity and richness, as well as an increased bacterial load, in dogs affected with *B. bronchiseptica* compared with healthy dogs. The Alcaligenaceae family and the *Bordetella* genus were overrepresented in diseased dogs. In the sequencing profile, about half of the diseased dogs were co-infected, the majority with *M. cynos*. Finally, a positive correlation was found between the bacterial composition of the LM for *B. bronchiseptica* and *M. cynos* at each taxonomic level and the corresponding qPCR or culture result.

In this study, the major phyla found in healthy dogs were the Proteobacteria, the Bacteroïdetes, the Actinobacteria and the Firmicutes. The same major phyla have already been reported in the LM of healthy dogs [28, 29, 31]. Despite their implication in CIRD-C, *B. bronchiseptica* and *M. cynos* are commensal bacteria found in the respiratory tract of healthy dogs [1, 4, 8]. In the present study, the amplicon sequencing technique detected *B. bronchiseptica* at very low level in 1 healthy dog, in which qPCR revealed a very low load. The absence of dysbiosis associated with the presence of *B. bronchiseptica* at a very low level in that dog, corroborates the fact that this bacterium is a commensal bacterium which is not always associated with lung disease [1, 4, 8]. The amplicon sequencing technique also detected *M. cynos* in low relative abundance in 3 of the healthy dogs, while qPCR results were negative. Since different aliquots from a same initial sample of BALF were used for qPCR and amplicon sequencing technique, a lack of homogeneity between the aliquots could explain this slight discrepancy.

Compared with healthy dogs, a dysbiosis was observed in the diseased dogs, with a shift in microbial populations as shown by a clear difference in the β-diversity. The Proteobacteria and the Tenericutes phylum were more abundant in the diseased dogs, logically reflecting an increased prevalence of *Bordetella* and *Mycoplasma.* The incapacity to show significant differences between healthy and diseased dogs at the species level was probably due to a lack of power in the statistical tests related to the low number of control dogs included in the study as well as to the high number of data (10 000 sequences per sample). Indeed, large dataset requires more severe corrections for multiple tests [39]. In dogs affected with *B. bronchiseptica* infection, in comparison with heathy dogs, the LM was composed in majority by only one or two bacteria, a finding that has also been reported in dogs with CAP [30]. In pneumonia in man, the dominant pathogenic strain also usually represents the majority of the detected sequences (74% or more) [24]; a low alpha-diversity and low richness reflecting the high predominance of one or two bacteria are also described together with an increased bacterial load [26]. In the present study, we observed identical modifications since the α-diversity and the richness were drastically lower and the bacterial load higher in diseased compared with healthy dogs. These modifications are supported by the ecological modeling of the LM proposed by Dickson et al. [26]. In healthy individuals, the bacterial communities found in the LM are mainly determined by the balance between immigration and elimination while in injured respiratory tract, the local growth conditions are altered creating a pressure across bacterial members and improving the reproduction rate of adapted bacteria which results in an increase in the bacterial load and a decrease in the richness and the diversity, together with the emergence of dominating bacteria [26].

The prevalence of bacterial co-infections in dogs affected with *B. bronchiseptica* found in this study by sequencing is quite elevated (40%) in comparison with data from the literature, where bacterial co-infections are reported in 7.69% to 53% of cases [1, 3, 6]. Reported co-infecting bacteria in CIRD-C also found in that study by sequencing included *M. cynos* [1], other *Mycoplasma* species [3, 6] and *Pseudomonas* sp. [8]. Other bacteria with a relative abundance > 5% that have been associated with pneumonia such as *Stenotrophomonas* sp., *Ureaplasma* sp., *Escherichia*-*Shigella* sp. in dogs [5, 30, 40–42], or *Elizabethkingia meningoseptica* in man [43] were found in that study. Although it is unclear if they are just colonizing or co-infecting bacteria, and if they could potentially play a role in CIRD-C. The high rate of co-infections in this study could be associated with the selection of the diseased dogs. Indeed, in CIRD-C, the disease is often self-limiting and resolves spontaneously within 2 weeks without complications [8] while co-infections are usually associated with more severe and chronic clinical signs [4]. The diseased dogs were referral cases with clinical signs for a median duration of 1 month. Higher bacterial co-infection rate could also be related to underlying viral infection [4, 8], which was not assessed in this study.

As previously reported and confirmed in this study, the qPCR is a very sensitive technique to diagnose *B. bronchiseptica* infection [1]. All infected dogs tested in this study had a positive qPCR result for *B. bronchiseptica* generally at moderate to very high load. The result of the culture was negative in 5/11 dogs which could partially be related to the fact that four of those dogs had recently been treated with antimicrobial drugs which may impair culture growth. Negative culture results have also already been described in dogs with *B. bronchiseptica* infection and could be associated with the sensitivity of the technique [1]. In man, it has been shown that the culture sensitivity in *Bordetella sp.* infections was lower than the PCR sensitivity [44]. In the present study, *B. bronchiseptica* was found by 16S rDNA amplicon sequencing in high amount in all the diseased dogs. The results of the amplicon sequencing at each taxonomic level were correlated with the Ct classes and the culture results. Such good agreement between positive culture results and 16S rDNA sequencing results has already been reported [30], with a high relative abundance of the taxa found by culture. Also, as already reported, some ubiquitous bacteria identified by culture were not found with the 16S rDNA amplicon sequencing which could be due to a mis annotation of the SILVA database or to a contamination of the culture which could lead to errors in culture-based antimicrobial drug selection [30]. Other co-infecting and/or colonizing bacteria were detected by the sequencing, showing that the 16S rDNA amplicon sequencing can be an interested technique to identified new potential pathogens. Moreover, the sequencing depicts the global bacterial population on the contrary of the qPCR and culture. Indeed, the qPCR is specific of the targeted sequence and is not useful to detect new bacteria [45]. Culture is quite challenging; some bacteria like *Mycoplasma* sp. requires specific culture conditions, some bacteria are unculturable and other bacteria are rare and slow growing and therefore may be missed [20]. The present study has some limitations. Firstly, qPCR Ct and culture results were not available in all dogs. Moreover, the qPCRs were performed on different type of materials (frozen or fresh, pellet or crude BALF). Some dogs were treated with antimicrobial drugs at the time of sampling which could have an impact on culture, qPCR and sequencing results. Culture results of BALF samples from healthy dogs were not available. We consider that such results are not essential since our study focuses on the evaluation of the 16S rDNA amplicon sequencing technique in diseased dogs, in a clinical context. Besides, we have a quite limited number of control dogs and in order to compare age-matched groups, we have selected a subpopulation of our diseased dogs for the comparison. Indeed, although in dogs the effect of aging has not been studied, in man, the LM has been reported to be different in young children of less than 3 years compared with adults [24]. Healthy dogs were not breed-matched with the diseased dogs. However, the breed impact on the LM seemed to be subtle [31]. No differences between the selection of diseased dogs and the rest of the diseased group were shown suggesting that the selection is representative of the diseased group.

In dogs with CIRD-C and *B. bronchiseptica* infection, there is a major dysbiosis of the LM, characterized by high bacterial load, low richness and diversity and increased abundance of *B. bronchiseptica*, in comparison with healthy dogs.

Co-infections, mostly with *M. cynos*, are frequent in CIRD-C dogs with *B. bronchiseptica* infection and could have an impact on the duration of the disease and the response to treatment.

The sequencing results highly correlated with results obtained by specific qPCR of *B. bronchiseptica* and *M. cynos* and culture of *B. bronchiseptica.* Therefore, 16S rDNA amplicon sequencing is reliable to identify potential causal bacterial microorganism involved in lung infectious diseases, to understand the global interaction between bacteria in the lung and could be useful to identify new species potentially involved in respiratory diseases in dogs.

In the future, with the development of 16S technologies, it could be interesting to include those analyses in the diagnostic work-up, mostly in dogs with a suspicion of lower airway infection, especially when the classical culture is negative or when there is no or only poor response to classical treatment. However, in such case, additional culture will still be needed to detect bacterial resistance to antimicrobial drugs.

## Supplementary information

**Additional file 1. Comparison between the subpopulations of diseased dogs selected or not for the comparison with healthy dogs.** Results of the TCC, DCC and all LM parameters comparison between the subpopulations of diseased dogs selected (*n* = 7) or not (*n* = 13) for the comparison with healthy dogs.

## Data Availability

All sample raw reads were deposited at the National Center for Biotechnology Information and are available under Bioproject ID PRJNA575149.

## Abbreviations

BAL: bronchoalveolar lavage
BALF: bronchoalveolar lavage fluid
CAP: community-acquired pneumonia
Ct: cycle threshold
CIRD-C: canine infectious respiratory disease complex
DCC: differential cell count
HOMOVA: homogeneity of molecular variance
LM: lung microbiota
OTU: operational taxonomic unit
PCA: principal component analysis
PCR: polymerase chain reaction
PCS: procedural control specimen
PERMANOVA: permutational analysis of variance
qPCR: quantitative polymerase chain reaction
TCC: total cell count
