# Supplementary material for: Analysis of the lung microbiota in dogs with Bordetella bronchiseptica infection and correlation with culture and quantitative polymerase chain reaction
Source: Vet Res. 2020 Mar 24;51:46. doi: 10.1186/s13567-020-00769-x (PMC7092585; doi:10.1186/s13567-020-00769-x)
Supplement: Supplementary file 1 — Additional file 1. Comparison between the subpopulations of diseased dogs selected or not for the comparison with healthy dogs. Results of the TCC, DCC and all LM parameters comparison between the subpopulations of diseased dogs selected (n = 7) or not (n = 13) for the comparison with healthy dogs. [file 13567_2020_769_MOESM1_ESM.docx]

**Additional file 1 Comparison between the subpopulations of diseased dogs selected or not for the comparison with healthy dogs.**

| Variable | | Subpopulation of diseased dogs selected to be compared with healthy dogs (*n* = 7) | Subpopulation of diseased dogs not selected to be compared with healthy dogs (*n* = 13) | *P*-value |
| --- | --- | --- | --- | --- |
| Clinical findings | Age (y) | 0.99 (0.81-1.02) | 0.51 (0.38-0.56) | <0.001^†^ |
|  | Gender (F/M) | 4/3 | 8/5 |  |
| BALF analysis | TCC (cell/µL) | 1300 (1040-3622) | 1780 (1391-3510) | 0.71^†^ |
|  | Macrophages (%) | 33 (15.8-47.2) | 49 (13-66) | 0.42^†^ |
|  | Neutrophils (%) | 48 (35.8-68.5) | 34 (21.5-77) | 0.85^†^ |
|  | Lymphocytes (%) | 9.5 (6.2-15.8) | 7 (3-10) | 0.20^†^ |
|  | Eosinophils (%) | 2 (1-6.8) | 1 (0-4.5) | 0.42^†^ |
| Lung microbiota parameters | β-diversity | / | / | 0.30^††^ |
|  | α-diversity (Inverse Simpson index | 1.56 (1.08-1.92) | 1.12 (1.08-1.98) | 0.88^†^ |
|  | Richness (chao index) | 69.1 (54-94.8) | 61.3 (37-98) | 0.64^†^ |
|  | Evenness (Simpson derived index) | 0.034 (0.027-0.038) | 0.045 (0.028-0.072) | 0.28^†^ |
|  | Bacterial load (log 16S rDNA copy numbers/mL) | 5.71 (5.47-5.92) | 5.79 (5.66-6.04) | 0.64^†^ |

BALF, bronchoalveolar lavage fluid; ^†^, Mann-Whitney tests; ^††^, permutational analysis of variance. The subpopulation of diseased dogs selected to be compared with healthy dogs corresponds to the dogs n°3, 9, 14, 15, 18, 19 and 20 in the Table 1.
